# Redesigning residency training with artificial intelligence: Implications for efficiency, quality, and workforce capacity

**DOI:** 10.1002/acm2.70875

**Published:** 2026-10-26

**Authors:** Laurence E Court, Beth Beadle, Comron J Hassanzadeh, Skylar S Gay, Tucker J Netherton, Rebecca M Howell, Adam D Melancon

**Affiliations:** ^1^ Department of Radiation Physics University of Texas MD Anderson Cancer Center Houston Texas USA; ^2^ Stanford University Stanford California USA; ^3^ Department of Radiation Oncology University of Texas MD Anderson Cancer Center Houston Texas USA

**Keywords:** AI‐based training, residency training

## Abstract

**Purpose:**

Workforce shortages and increasing clinical demands in Radiation Oncology and Medical Physics present challenges for maintaining high‐quality residency training. This work presents a conceptual framework to evaluate the potential role of artificial intelligence (AI) in improving efficiency, scalability, and quality of residency education.

**Methods:**

Current teaching and learning modalities were categorized, and their relative contributions to training were estimated. Potential AI applications were mapped to these modalities, and the proportion of training that could be supported by AI was assessed. A simple conceptual model incorporating training time, AI‐deliverable components (i.e., those potentially supported through direct interaction with AI systems), and dependence on direct human teaching was used to illustrate the potential impact on instructional effort. The potential effect of AI on educational quality was qualitatively evaluated, as well as limitations of AI for routine educational integration.

**Results:**

AI applications were identified across multiple domains, including workflow‐integrated feedback, simulation‐based training, adaptive learning, and automated assessment. The largest potential impact was estimated in workflow‐integrated, simulation‐based, and self‐directed learning environments. While reductions in direct human teaching effort were estimated to be modest, AI‐enabled approaches were estimated to achieve substantial improvements in training quality, including increased feedback frequency, enhanced support for deliberate practice, and greater exposure to rare or high‐risk scenarios.

**Conclusions:**

AI has the potential to enhance residency training by improving consistency, scalability, and educational quality. In the context of workforce constraints, the primary benefit of AI may be to augment training efficiency and reduce reliance on limited faculty resources while preserving the essential role of clinical experience.

## 1 INTRODUCTION

Radiation therapy is a cornerstone of cancer care, with demand increasing globally due to rising cancer incidence and expanding treatment indications.^1^ Concurrently, workforce shortages in both Radiation Oncology and Medical Physics are well‐documented across high‐ and low‐resource settings.^2^, ^3^, ^4^ Increasing clinical complexity, including advanced treatment techniques, adaptive workflows, and stringent quality requirements, limits the capacity of departments to expand training programs or devote faculty time to education.

Residency training in Radiation Oncology and Medical Physics relies heavily on experiential learning within routine clinical workflows, where competency is closely linked to case volume, repetition, and supervision.^5^ While this model has been successful, it is inherently variable and may provide inconsistent opportunities for structured feedback, deliberate practice, and exposure to uncommon scenarios. Assessment is often episodic and subjective, and patient safety considerations appropriately limit opportunities for trainees to learn through failure or experimentation in real clinical environments.

Artificial intelligence (AI), including machine learning, generative models, adaptive learning systems, and clinical decision‐support tools, has the potential to address many of these limitations. Emerging evidence suggests that AI‐enabled educational approaches can improve clinical competence and training outcomes, while broader applications are being explored across multiple medical and surgical specialties.^6^, ^7^, ^8^, ^9^, ^10^, ^11^, ^12^, ^13^, ^14^, ^15^, ^16^

At the same time, successful integration of AI into residency education requires careful consideration to ensure educational quality is maintained and unintended consequences are avoided.^17^ A key limitation of traditional residency training is the difficulty of consistently supporting deliberate practice, defined as repetitive task performance accompanied by immediate feedback and opportunities for refinement.^18^ Simulation‐based training incorporating deliberate practice has been shown to produce superior skill acquisition compared to traditional clinical education.^19^ In Radiation Oncology and Medical Physics, many core competencies depend on iterative refinement, yet feedback is frequently constrained by workflow demands. AI‐enabled systems may overcome these barriers by providing scalable, standardized, and high‐frequency feedback within both simulated and clinical learning environments.

In this work, we present a conceptual framework describing how AI could influence residency training in Radiation Oncology and Medical Physics. We characterize current teaching and learning modalities, map potential AI applications onto these activities, and explore their potential impact on teaching effort, educational quality, and future program design. Although focused on Radiation Oncology and Medical Physics, many of the challenges addressed are shared across other specialties, suggesting broader applicability of the framework. The estimates presented are intended to be illustrative and evidence‐informed rather than predictive.

## 2 HOW WE TEACH TODAY

Current residency training in Radiation Oncology and Medical Physics spans a range of clinical, workflow‐integrated, simulation‐based, didactic, self‐directed, and assessment‐driven learning activities (Table 1). Clinical experience remains the dominant component of training, while simulation, didactic education, and self‐directed study contribute supporting roles. The distributions shown in Table 1 highlight where educational effort is currently concentrated and provide context for evaluating the potential role of AI in residency training.

**TABLE 1:** Teaching / learning distribution in residency (estimates of % of learning exposure, based on authors’ experience).

Category	Subtype / examples	Radiation oncology (%)	Medical physics (%)
Clinical (active)	Patient care, contouring, OTVs, linac QA, planning	45–60	30–45
Clinical (passive)	Shadowing, observation, tumor boards	5–10	3–8
Workflow‐integrated	Chart review, plan eval, on‐the‐fly teaching	10–20	10–20
Simulation / lab‐based	Phantom work, measurements, treatment planning exercises	3–8	15–25
Didactic (large group)	Lectures, conferences	5–10	5–10
Didactic (small group)	Case discussions, tutorials	5–10	5–10
Self‐directed	Reading, board preparation, guidelines (e.g., ASTRO, AAPM)	10–20	10–20
Assessment‐driven	Exams, mock orals, competency reviews	2–5	3–7

## 3 HOW AI COULD BE USED IN TRAINING

A common challenge is the availability of timely, objective feedback needed to support deliberate practice and consistent competency development. Increasing clinical demands and evolving work models may further limit opportunities for direct observation and experiential learning. Potential AI applications in residency education can be grouped according to their primary educational function (Table 2). These categories provide a framework for understanding where AI may contribute across the major domains of residency training.

**TABLE 2:** Core categories where AI can aid training/learning.

Category	Primary function	Example in residency
knowledge & personalization	Learning content	Adaptive board prep
Simulation & skills	Practice	AI contouring / brachytherapy trainer
Clinical decision support	Learning in care	Plan recommendations
Workflow‐integrated	Real‐time feedback	QA alerts + teaching
Assessment & feedback	Evaluation	Autocontour scoring
Teaching augmentation	Faculty support	AI‐generated cases
Safety & error learning	Learning from mistakes	Incident pattern detection

Table 3 maps major training modalities to potential AI roles. The broad distribution of applications suggests that AI may augment learning across clinical, didactic, simulation‐based, and self‐directed settings, with particularly strong opportunities in workflow‐integrated learning, simulation, and adaptive educational support

**TABLE 3:** Mapping teaching style to AI roles.

Teaching / learning style	Where It Occurs	Primary AI Roles	Example in Residency (Rad Onc / Physics)
Clinical (active)	Patient care, QA, planning	Clinical decision support (educational)workflow‐integrated learning	Real‐time DVH deviation analysis with suggested constraint adjustments; comparison to institution‐specific high‐quality plan library
Clinical (passive)	Shadowing, observation	Teaching augmentation knowledge support (just‐in‐time)	AI “second voice” explaining what the attending is doing during cases
Workflow‐integrated learning	Real‐time clinical work	Workflow‐integrated learning (real‐time feedback), error detection & safety learning	Automated contour scoring against consensus atlas with spatial error heatmaps, Plan QA: AI flags deliverability issues and links to prior similar failure cases from incident learning systems
Simulation / lab‐based	Phantom work, planning exercises	Simulation & skills training assessment & feedback	Deliberate practice loop: generate contours and plan → AI scoring (contour fidelity, dosimetry, conformity, OAR sparing) → targeted feedback → repeat; virtual brachytherapy; radiopharmaceutical therapy simulation, Injected error scenarios (e.g., MLC misconfiguration, contour omission) based on real incident datasets; virtual linac environments
Didactic (large group)	Lectures	Knowledge acquisition & personalization teaching augmentation	AI‐generated summaries of ASTRO or AAPM guidelines; lecture Q&A assistants
Didactic (small group)	Case discussions, tutorials	Teaching augmentation clinical decision support (educational layer)	AI suggests differential plans during case discussions; generates probing questions
Self‐directed learning	Reading, board prep	Knowledge acquisition & personalization assessment & feedback	Adaptive question banks, personalized study plans, instant explanations
Assessment‐driven learning	Exams, evaluations	Assessment & feedback automation	Longitudinal skill curves (e.g., contour DSC vs time, plan quality metrics) with automated identification of plateau or regression
(Cross‐cutting)	Across all settings	Error detection & safety‐based learning cognitive amplification	AI flags near‐misses and turns them into teaching cases; supports reasoning rather than just answers

Representative applications include AI‐assisted contouring with immediate comparison to expert benchmarks, real‐time treatment planning feedback incorporating comparisons with high‐quality historical plans, and simulated error‐based learning environments derived from incident learning systems. Together, these examples illustrate how AI could support deliberate practice, feedback, and competency development across multiple components of residency training.

Table 4 estimates the proportion of each training modality that could potentially be supported through direct interaction with AI systems. The highest levels of AI‐deliverable content are observed in simulation‐based, self‐directed, and workflow‐integrated learning, whereas activities involving direct patient care remain substantially dependent on human judgment and responsibility. Figure 1 provides a visual representation of the potential AI‐supported fraction of training across the major educational domains.

**TABLE 4:** Estimated % of Training Potentially Deliverable via Direct AI Interaction.

Category	% Range	Explanation
Clinical (active)	5–30	Core hands‐on activities (patient interaction, procedures, responsibility) cannot be replaced. However, AI can support contouring, planning, QA review, and decision reasoning, especially in a supervised or parallel learning mode.
Clinical (passive)	60–90	Observation can be significantly reduced or enhanced with AI‐guided walkthroughs, annotated cases, and “explain‐as‐you‐go” systems, often superior to traditional shadowing.
Workflow‐integrated	40–70	High potential: much of this learning is already informal and unstructured. AI can provide real‐time feedback, plan evaluation, and teaching prompts, making it one of the most “AI‐compatible” domains.
Simulation / lab‐based	60–90	Strongest area for AI substitution. Many tasks (treatment planning, QA scenarios, error simulation) can be fully virtualized or AI‐driven, especially in medical physics training.
Didactic (large group)	50–80	Lectures can be replaced or supplemented by AI tutors, adaptive modules, and interactive Q&A. Limitation: loss of shared experience and expert narrative framing.
Didactic (small group)	40–70	AI can simulate case discussions and Socratic questioning. However, human nuance, debate, and mentorship still add value, especially in radiation oncology.
Self‐directed	70–95	Nearly ideal for AI: adaptive learning, question generation, summarization, and personalized pacing. This is likely the most immediately transformable domain.
Assessment‐driven	50–85	Many components (knowledge testing, contour evaluation, plan assessment) can be automated. Limits include high‐stakes exams and professional judgment assessments.

**FIGURE 1:**
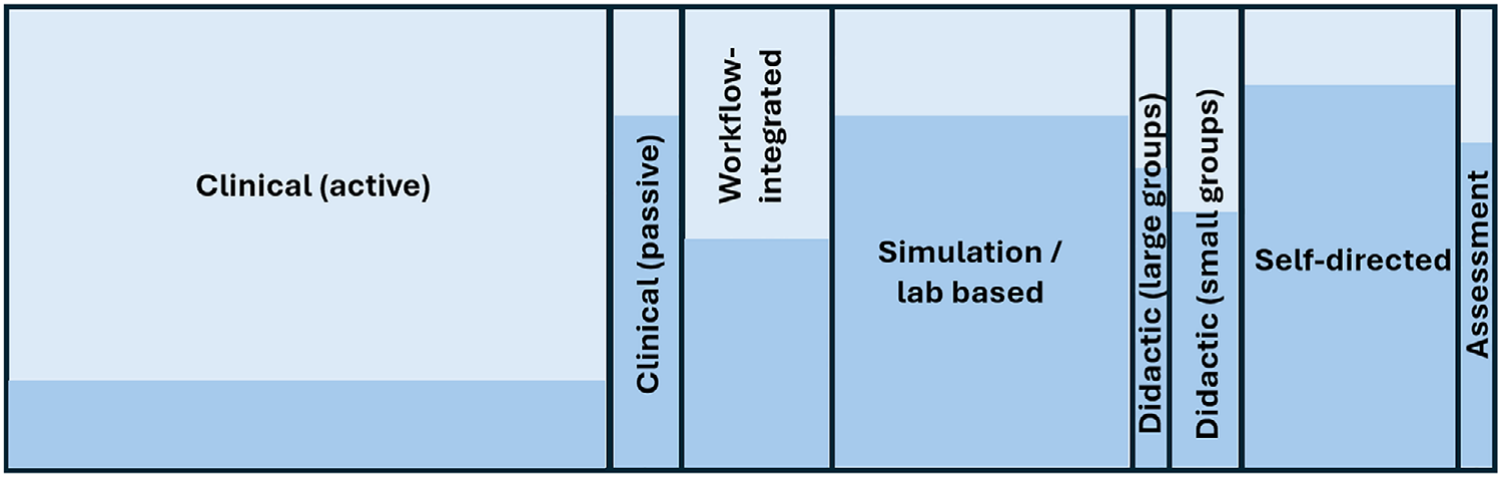
Distribution of time spent in different categories of learning (area of each box), and the fraction of that that could be supplemented by AI (dark blue area).

## 4 IMPACT OF AI TRAINING ON TRAINING EFFICIENCY

Now that we have an idea of where AI could supplement the residency experience, we can examine the potential impact of AI on training efficiency. Table 5 suggests that the impact of AI on teaching effort is driven by both AI compatibility and the proportion of training devoted to a given activity. The greatest opportunities occur in clinical and workflow‐integrated learning, while self‐directed learning contributes less to overall efficiency despite high AI compatibility. Overall, AI appears more likely to redistribute teaching effort than substantially reduce the need for human educators.

**TABLE 5:** Modeled reduction in direct human teaching time.

Category	T (%)	A (%)	H (%)	Total: T × A × H (%)	Explanation
Clinical (active)	45–60	10–30	70–95	3–17	High T and H, but limited AI substitutability keeps total moderate
Clinical (passive)	5–10	60–90	60–90	2–7	AI can replace much explanatory value of shadowing
Workflow‐integrated	10–20	40–70	50–80	2–11	Strong candidate: moderate T × high A × moderate‐high H
Simulation / lab‐based	3–8	60–90	40–75	1–5	Very AI‐compatible, but smaller share of total training
Didactic (large group)	5–10	50–80	80–100	2–8	High replaceability of repetitive teaching
Didactic (small group)	5–10	40–70	85–100	2–7	Still human‐valued, but partially substitutable
Self‐directed	10–20	70–95	10–30	1–6	High AI utility, but low baseline human teaching limits impact
Assessment‐driven	2–5	50–85	50–85	1–4	Automation possible, but high‐stakes elements remain human

*Note*: T (% of training), A (% AI‐deliverable), H (% human teaching dependence).

## 5 IMPACT OF AI TRAINING ON QUALITY

Table 6 suggests that the greatest impact of AI may be on educational quality rather than efficiency. The largest gains are anticipated where AI can enable deliberate practice, frequent feedback, personalized learning, and exposure to rare or error‐based scenarios that are difficult to achieve consistently in routine clinical training.

**TABLE 6:** AI‐enabled quality gains by teaching category.

Category	AI‐enabled quality gain (relative to typical training)	Not feasible in routine clinical setting?	Explanation
Clinical (active)	Low–moderate	Partial	AI can enhance reasoning (e.g., plan trade‐offs, contour feedback), but real patient care constraints limit experimentation, repetition, and failure‐based learning.
Clinical (passive)	High	Yes	AI can turn passive observation into actively guided learning with explanations, annotations, and structured reflection, often superior to traditional shadowing.
Workflow‐integrated	Moderate–high	Yes (partially)	AI can provide real‐time, consistent micro‐feedback during routine work, something that is currently variable and often absent due to time pressure.
Simulation / lab‐based	Very high	Yes	AI enables deliberate practice, error simulation, and unlimited repetition, far beyond what machine time, staffing, and clinical priorities allow.
Didactic (large group)	Moderate	No (mostly)	AI improves personalization and interactivity, but core content delivery is already feasible; gains are incremental rather than transformative.
Didactic (small group)	Moderate	Partial	AI can simulate discussion and questioning, but human nuance and debate are difficult to replicate fully. Gains mainly in scalability and consistency.
Self‐directed	High–very high	Yes (partially)	AI enables adaptive learning, instant feedback, and targeted remediation, which are not achievable with static resources alone.
assessment‐driven	High	Yes (partially)	AI enables continuous, objective, granular assessment and feedback, which is difficult to achieve with episodic, human‐driven evaluations.

## 6 POTENTIAL RISKS OF AI IN RESIDENCY TRAINING

While AI offers significant potential benefits for residency training, it also introduces new risks.^7^, ^8^, ^20^, ^21^ These risks vary across training modalities and are summarized in Table 7. A recurring concern is cognitive offloading, whereby trainees rely on AI‐generated feedback and content rather than developing independent reasoning. Many of the highest‐risk domains correspond to areas of extensive AI integration, underscoring the importance of appropriate supervision and implementation strategies that ensure AI augments rather than replaces core skill development.

**TABLE 7:** Potential risks of AI in residency training.

Category	Potential risk level	Primary risks	Explanation
Clinical (active)	Moderate–high	Over‐reliance on AI, reduced hands‐on experience	AI support in contouring, planning, and QA may reduce opportunities for trainees to develop independent technical and clinical judgment if not carefully supervised.
Clinical (passive)	Low–moderate	Reduced exposure to real clinical dynamics	AI‐guided observation may not fully replicate the interpersonal, multidisciplinary, and situational aspects of real clinical interactions.
Workflow‐integrated	Moderate	Cognitive offloading, reduced critical thinking	Real‐time AI feedback may lead trainees to rely on AI outputs rather than actively engaging in independent analysis, particularly in high‐throughput clinical settings.
Simulation / lab‐based	Moderate	Limited realism, overconfidence	AI‐enabled simulation may not fully capture the variability and complexity of real clinical environments, potentially leading to overconfidence or gaps in practical skills.
Didactic (large group)	Low–moderate	Loss of expert synthesis, reduced shared learning	Replacement of lectures with AI‐driven content may reduce exposure to expert interpretation, narrative framing, and shared learning experiences among trainees.
Didactic (small group)	Moderate	Reduced human interaction, loss of nuance	AI‐facilitated discussions may lack the depth of human‐led debate, mentorship, and real‐time adaptation to learner needs.
Self‐directed	Moderate–high	Knowledge gaps, bias, over‐reliance	AI‐generated content may contain inaccuracies or biases, and heavy reliance on AI for learning may limit development of foundational knowledge and independent study habits.
Assessment‐driven	Moderate–high	Over‐standardization, gaming metrics	Automated assessment may incentivize optimization for measurable metrics rather than deeper understanding and may miss higher‐order competencies such as judgment and professionalism.

## 7 PRIORITY AREAS FOR AI DEVELOPMENT IN RESIDENCY TRAINING

Ongoing workforce shortages in Radiation Oncology and Medical Physics highlight the need for residency training models that are both scalable and educationally effective. The framework presented in this work suggests that not all AI applications are likely to have equal impact. The greatest opportunities appear in areas where feedback is currently limited, faculty time is constrained, or exposure to important clinical scenarios is inconsistent. Collectively, the analyses presented in Tables 4, 5, 6, 7 suggest three high‐priority areas for future AI development in residency education.

1. **Real‐Time Workflow‐Integrated Feedback Systems**. AI systems embedded within routine clinical workflows could provide immediate educational feedback during activities such as contouring, treatment planning, image guidance, and quality assurance. Rather than functioning solely as clinical decision‐support tools, these systems could provide explanations, identify opportunities for improvement, and reinforce best practices while residents perform routine clinical work. By delivering consistent, real‐time feedback, workflow‐integrated AI has the potential to reduce variability arising from faculty availability and transform everyday clinical activities into richer learning experiences. Importantly, these systems are intended to augment, rather than replace, expert supervision.
2. **AI‐Driven Simulation, Deliberate Practice, and Safety Training**. Simulation represents one of the most promising applications of AI in residency education. AI‐enabled simulation environments could support deliberate practice through unlimited repetition, adaptive difficulty, automated feedback, and objective performance assessment. Beyond procedural and technical skills, AI could generate realistic error scenarios and near misses derived from incident learning systems, allowing residents to explore clinically important failure modes within a safe educational environment. Such experiences are difficult to provide consistently through routine patient care but are well aligned with established principles of simulation‐based education and deliberate practice. Together, AI‐enabled simulation and error‐based learning may substantially improve both technical competency and patient safety awareness.
3. **Continuous Assessment and Adaptive Personalized Learning**. AI has the potential to transform assessment from an episodic activity into a continuous educational process. Longitudinal tracking of resident performance could identify learning gaps earlier, monitor competency development over time, and support competency‐based progression. At the same time, adaptive learning systems could use these assessment data to personalize educational content, generate targeted questions, recommend additional practice, and provide immediate feedback tailored to individual needs. By tightly coupling continuous assessment with personalized learning, AI could create an iterative learning cycle that supports more efficient remediation while reducing the administrative burden associated with traditional trainee evaluation.

Although these three priorities address different aspects of residency education, they are complementary. Workflow‐integrated feedback supports learning during routine clinical practice, simulation provides opportunities for deliberate practice outside the clinic, and continuous assessment enables personalized learning over time. Together, these approaches have the potential to shift residency education from a predominantly opportunistic model toward one that is more structured, adaptive, and learner‐centered.

## 8 HOW WILL AI TRAINING AFFECT RESIDENCY PROGRAM DESIGN

The integration of AI has the potential to reshape residency training in Radiation Oncology and Medical Physics by reducing reliance on clinical volume as the primary mechanism for skill acquisition. AI‐enabled approaches, including simulation, real‐time feedback, and adaptive learning, may support more structured and deliberate learning outside routine clinical workflows, with greater emphasis on feedback, error‐based learning, and targeted remediation. However, clinical experience and direct faculty mentorship will remain fundamental components of residency education.

AI is also being increasingly integrated into clinical practice, including applications in contouring, treatment planning, and quality assurance. As these tools become more widely adopted, trainee participation may shift away from some manual tasks toward oversight, interpretation, and clinical decision‐making. The evolution of clinical AI and educational AI are therefore likely to occur in parallel, reinforcing the need for training programs that emphasize competencies within AI‐augmented clinical environments.

## 9 DISCUSSION

Artificial intelligence has the potential to address several longstanding challenges in residency training in Radiation Oncology and Medical Physics, particularly in the setting of increasing clinical demand and workforce shortages. In this work, we developed a conceptual framework describing how AI may influence teaching effort, educational quality, and future residency program design. The framework identifies where AI is most likely to provide educational value and where human expertise will remain essential. Overall, our analysis suggests that AI is likely to improve the quality, consistency, and scalability of residency education to a greater extent than it reduces faculty teaching effort. To our knowledge, this is the first framework to consider training modality, AI applicability, teaching efficiency, educational quality, and educational risk within a single model.

The framework highlights three particularly promising areas for future development: workflow‐integrated feedback during routine clinical practice, AI‐enabled simulation supporting deliberate practice and safety training, and continuous assessment linked to adaptive personalized learning. Together, these approaches have the potential to shift residency education from a predominantly opportunistic model, in which learning depends largely on available clinical experiences, toward one that is more structured, individualized, and continuously supported. Such changes may allow faculty to devote proportionally more effort to mentorship, professional development, and higher‐order clinical reasoning rather than repetitive instruction.

Successful implementation will require careful attention to educational quality. AI should augment rather than replace independent clinical reasoning, and training programs must guard against automation bias, cognitive offloading, and over‐reliance on AI‐generated recommendations. Educational systems should therefore be designed to promote critical thinking, provide transparent feedback, and ensure that core competencies can be demonstrated independently of AI assistance. Emerging concepts such as “never‐skilling,” in which excessive reliance on AI prevents acquisition of fundamental competencies, further emphasize the need for thoughtful educational design rather than simple technology adoption.

This framework focuses primarily on how AI may influence the trainee experience. Important related topics—including AI‐assisted curriculum development, educational content generation, faculty support, and formal instruction about the safe and effective use of AI itself—were beyond the scope of this work but represent important directions for future research. In addition, the quantitative estimates presented throughout this paper are intentionally illustrative rather than predictive. They are intended to stimulate discussion and provide a structured basis for future empirical evaluation rather than precise forecasts of educational impact.

Although this framework is presented from the perspective of residency training in the United States, many of the underlying educational challenges are shared internationally. AI‐enabled educational approaches may therefore have broad applicability, particularly in regions where workforce shortages, limited educational resources, or geographic barriers constrain access to expert mentorship. Differences in infrastructure, regulation, and technology availability will influence implementation, but the educational principles described here are likely to remain broadly relevant.

In conclusion, AI is unlikely to replace clinical training or expert educators. Its greatest value may instead lie in enabling more consistent feedback, deliberate practice, objective longitudinal assessment, and personalized educational experiences while preserving the essential role of clinical experience, faculty mentorship, and professional judgment. As AI becomes increasingly integrated into both clinical practice and medical education, residency programs have an opportunity not simply to adopt new technologies, but to rethink how expertise is developed and how the next generation of Radiation Oncologists and Medical Physicists is trained.

## AUTHOR CONTRIBUTIONS

All authors contributed to the implementation of the research, data collection, the analysis of the results, and writing the manuscript. **Beth Beadle**: Implementation of the research, data collection, the analysis of the results, and writing the manuscript. **Laurence E Court**: Implementation of the research, data collection, the analysis of the results, and writing the manuscript. **Skylar S Gay**: Implementation of the research, data collection, the analysis of the results, and writing the manuscript. **Comron J Hassanzadeh**: Implementation of the research, data collection, the analysis of the results, and writing the manuscript. **Rebecca M Howell**: Implementation of the research, data collection, the analysis of the results, and writing the manuscript. **Adam D Melancon**: Implementation of the research, data collection, the analysis of the results, and writing the manuscript. **Tucker J Netherton**: Implementation of the research, data collection, the analysis of the results, and writing the manuscript.

## CONFLICT OF INTEREST STATEMENT

The Radiation Planning Assistant project has been funded by the NI/NCI, Varian Medical Systems, Cancer Prevention Research Institute of Texas, Wellcome Trust and the University of Texas MD Anderson Cancer Center.

## FUNDING INFORMATION

The authors have nothing to report.

## ETHICAL APPROVAL

Not human‐subjects research.

## Data Availability

All data is included in the manuscript.
